# Anti-Leukemic Effects of *Idesia polycarpa* Maxim Branch on Human B-Cell Acute Lymphoblastic Leukemia Cells

**DOI:** 10.3390/cimb45050257

**Published:** 2023-05-04

**Authors:** Chan-Seong Kwon, Ji-Eun Lee, Byeol-Eun Jeon, Ye-Rin Woo, Yun-Seo Kim, Jae-Woo Kim, Chae-Jin Park, Seo-Yun Jang, Sang-Woo Kim

**Affiliations:** 1 Department of Integrated Biological Science, Pusan National University, Busan 46241, Republic of Korea; 2 Department of Biological Sciences, Pusan National University, Busan 46241, Republic of Korea

**Keywords:** B-cell acute lymphoblastic leukemia, *Idesia polycarpa* Maxim branch, anti-leukemic effect, differentiation, glucocorticoid resistance

## Abstract

Patients with pediatric B-cell acute lymphoblastic leukemia (B-ALL) have a high survival rate, yet the prognosis of adults and patients with relapsed/refractory disease is relatively poor. Therefore, it is imperative to develop new therapeutic strategies. Here, we screened 100 plant extracts from South Korean Flora and investigated their anti-leukemic effect using CCRF-SB cells as a B-ALL model. The top cytotoxic extract identified in this screening was the *Idesia polycarpa* Maxim. branch (IMB), which efficiently inhibited the survival and proliferation of CCRF-SB cells, while having minimal to no impact on normal murine bone marrow cells. Mechanistically, the IMB-induced proapoptotic effect involves the increase of caspase 3/7 activity, which was shown to be associated with the disruption of the mitochondrial membrane potential (MMP) through the reduction in antiapoptotic Bcl-2 family expression. IMB also promoted the differentiation of CCRF-SB cells via the upregulation of the expression of differentiation-related genes, PAX5 and IKZF1. Given that resistance to glucocorticoid (GC) is often found in patients with relapsed/refractory ALL, we investigated whether IMB could restore GC sensitivity. IMB synergized GC to enhance apoptotic rate by increasing GC receptor expression and downmodulating mTOR and MAPK signals in CCRF-SB B-ALL cells. These results suggest that IMB has the potential to be a novel candidate for the treatment of B-ALL.

## 1. Introduction

Acute lymphoblastic leukemia (ALL) arises from an abnormal proliferation of lymphocyte cells, which have not fully matured. ALL is divided into B-cell acute lymphoblastic leukemia (B-ALL) and T-cell acute lymphoblastic leukemia (T-ALL); 75% of ALL patients are diagnosed with B-ALL. Moreover, B-ALL is mostly found in children and consists of various subtypes based on mutations, such as non-random chromosomal rearrangements, aneuploidy, gene deletions, and amplifications [1,2].

Treatment of B-ALL can be divided into remission induction therapy and post-remission therapy. Induction therapy aims to eliminate leukemia cells detected in the body using chemotherapeutic drugs such as Vincristine, Prednisolone, Daunorubicin, and L-asparaginase (VPDL). Post-remission therapy consists of reinforcement/consolidation and maintenance therapy. This regimen is given to eliminate the remaining long-term leukemia cells [3,4,5,6]. With these standard treatments, the 5-year survival rate in children is as high as 90%, while in adults it is less than 45%, thereby showing an inferior prognosis in adults than in children.

Glucocorticoids (GC) are steroidal drugs mainly used to treat multiple myeloma (MM), chronic lymphocytic leukemia (CLL), and ALL. GC is involved in apoptosis and growth arrest of lymphoid malignant cells by inducing GR-mediated transcription factor activation and repression following binding to the glucocorticoid receptor (GR) [7,8]. GC resistance frequently occurs in malignant lymphocytes, and the mechanisms that induce GC resistance are well-studied. In GC-resistant cells, the downregulation of GRα expression and alteration in downstream signaling pathway genes are observed [7,8]. Recently, it was found that PAX5 and IKZF1: major regulators of differentiation in B-lymphocytes, modulate GR expression [9]. Further, alterations in the mitogen-activated protein kinase (MAPK) signaling pathway have been revealed in samples from patients with GC-resistant B-ALL. The previous report confirmed the activation of ERK, a MEK1/2 target, and an increase in p-ERK levels [10]. In addition, ALL with hyperactivity in the PI3K/PTEN/Akt/mTOR signaling pathway showed leukocyte hyperplasia and GC resistance [7,11].

Despite these efforts, GC resistance remains one of the biggest obstacles to the improvement of prognosis in patients with ALL; 10–30% of ALL patients show resistance to GC. Therefore, it is imperative to develop strategies that can restore GC sensitivity to improve the prognosis of patients with ALL.

Most of the drugs currently in clinical use have been derived from natural products. Commonly used drugs such as penicillin, tetracycline, rapamycin, and doxorubicin all originated from natural products [12]. Natural substances have diverse chemical structures, and many natural substances with bioactive components have not yet been identified [12]. For this reason, natural products are still being tested to search for novel medicinal properties. Indeed, the anticancer drug Paclitaxel (Taxol^®^) was developed from the Pacific yew tree through a natural product research program conducted by the US National Cancer Institute [13].

In this study, 100 species of native plants used in traditional medicine in Korea were screened to search for potential candidates for B-ALL treatment. We found extracts of *Idesia polycarpa* Maxim. branch (IMB), commonly called the wonder tree, exhibited an anti-leukemic effect. *Idesia polycarpa* Maxim. is a plant native to Korea, China, and Japan, which has been traditionally used as an herb due to its anti-inflammatory, antioxidant, and anticancer effects from the leaves and fruits. However, *Idesia polycarpa* Maxim. has not been tested in the treatment of leukemia. In the present study, we investigated the anti-leukemic effect of IMB using B-ALL cells. We found that IMB induces apoptosis by modulating the expression of Bcl-2 family members and the activity of MAPK and mTOR signaling pathways. Our data also demonstrated that IMB could be used to overcome GC resistance in B-ALL, suggesting that IMB may be a novel therapeutic agent in the treatment of this disease.

## 2. Materials and Methods

### 2.1. Preparation of Plant Extracts

Plant extracts used in this research were obtained from The Korea Plant Extract Bank at the Korea Research Institute of Bioscience and Biotechnology (Daejeon, Korea).

*Idesia polycarpa* Maxim (KPM051-072) is deposited in The Korea Plant Extract Bank at the Korea Research Institute of Bioscience and Biotechnology (Daejeon, Korea).

### 2.2. Cell Culture, Reagents, and Antibodies

CCRF-SB, a human ALL cell line, was cultured in RPMI-1640 medium (Hyclone, Logan, UT, USA) supplemented with 10% fetal bovine serum (FBS; Hyclone, QLD, Australia), 1% L-glutamine, 1% N-2-hydroxyethyl piperazine-N′-2-ethane sulfonic acid (HEPES) buffer, and 1% penicillin/streptomycin at 37 °C in a 5% CO_2_ incubator. Normal bone marrow cells isolated from wildtype C57BL/6 mice were cultured in RPMI-1640 medium with 10% FBS (Hyclone).

The chemicals used are as follows: dexamethasone (Sigma; St. Louis, MO, USA; 50-02-2), wortmannin (Sigma; St. Louis, MO, USA; W1628), and rapamycin (Santa Cruz Biotechnology; Dallas, TX, USA; sc-3504).

The primary antibodies against Bcl-2 (Santa Cruz Biotechnology; Dallas, TX, USA; sc-7381), Mcl-1 (Santa Cruz Biotechnology; Dallas, TX, USA; sc-819), p-AKT, p-4EBP1 14 (CST-9459) [S473; Cell Signaling Technology (CST); Danvers, MA, USA; 9271], p-4EBP1 (CST; 9459), p-ERK 1/2 (CST; 9101), and β-actin (Santa Cruz Biotechnology; Dallas, TX, USA; sc-4778) were used for western blots. HRP-conjugated anti-rabbit and anti-mouse secondary antibodies were from Bethyl Laboratories, Inc. (A120-101p and A90-116p-33; Montgomery, TX, USA).

### 2.3. Measurement of Cell Viability

The CellTiter 96 AQueous MTS assay was used to determine the cytotoxicity of IMB, according to the manufacturer’s instructions (Promega, Madison, WI, USA). Briefly, CCRF-SB and normal mouse bone marrow cells were plated at a density of 4 × 10^4^ cells and 5 × 10^5^ cells per well, respectively, in 96-well cell culture microplates and treated with IMB (0, 60, 80, or 100 µg/mL) for 24 h, followed by the addition of 30 μL of MTS reagent in the wells and incubation at 37 °C for 4 h. To test the combinatory effects, CCRF-SB cells were treated with IMB (0, 40, 50, or 60 µg/mL) and/or dexamethasone (0, 10, 100, or 1000 nM) for 48 h or 72 h. Absorbance was measured at 450 nm on a GloMax^TM^ Microplate multimode reader (Promega).

### 2.4. Apoptosis Assays

Cells were seeded at a density of 8 × 10^5^ cells per well in 12-well plates and treated with IMB (100 µg/mL) for 24 h. The apoptosis rate was analyzed using flow cytometry (BD FACSAria™ Fusion Flow Cytometer, BD Biosciences, Becton Drive Franklin Lakes, NJ, USA) after staining with propidium iodide (BD Biosciences, Franklin Lakes, NJ, USA).

### 2.5. Analysis of Caspase 3/7 Activity and Mitochondrial Membrane Potential

Caspase 3/7 assay was performed as previously described [14]. To confirm caspase 3/7 activity in human B-ALL cells, CCRF-SB was seeded at a density of 1 × 10^6^ cells per well in 12-well plates and treated with IMB (100 µg/mL) for 24 h. Caspase-Glo 3/7 Assay reagent (G8090; Promega, Madison, WI, USA) was added, and the cells were incubated at RT for 1 h, followed by luminescence measurement using GloMax^TM^ Microplate multimode reader (Promega). The mitochondrial membrane potential (MMP) was performed as previously described [15]. MMP was analyzed using fluorescence microscopy and FACS after treatment with IMB (100 µg/mL) for 24 h, followed by incubation with JC-1 (5,5′,6,6′-tetrachloro-1,1′,3,3′-tetraethylbenzimidazolylcarbocyanine iodide; Abnova, Taipei, Taiwan) dye for 30 min at 37 °C.

### 2.6. Western Blot Analysis

To perform Western blot analysis, CCRF-SB cells were seeded in a 12-well plate at 1.0 × 10^6^ cells per well and treated with IMB (100 µg/mL) for 24 h, or (40 µg/mL) for 48 h or 72 h, followed by lysis in Igepal ca-630 np 40 buffer (9002-93-1, Sigma, St. Louis, MO, USA) with 1 mM Na-vanadate, 50 mM β-glycerophosphate disodium salt, β-mercaptoethanol (142 mM; BioWORLD, Irving, TX, USA), ProteaseArrestTM (G-Biosciences; St. Louis, MO, U.S.A.), and EDTA (5 mM; G-Biosciences). Samples were boiled at 100 °C for 10 min in sample buffer, loaded into the polyacrylamide gels, and transferred onto Immobilon-P Transfer membranes, followed by blocking in 1% bovine serum albumin (BSA; MP Biomedicals, Santa Ana, CA, USA) and probing with primary antibodies at 4 °C overnight. After washing three times with Tris-buffered saline containing Tween 20 (TBS-T) for 5 min each, the membranes were probed with anti-mouse/rabbit secondary antibodies for 1 h at room temperature. After washing three times with TBS-T for 10 min each, the membranes were exposed to a chemiluminescent substrate (EzWestLumi plus (ATTO, Tokyo, Japan)), and the protein bands were visualized using a Luminograph II (ATTO).

### 2.7. Cell Proliferation Assay

The trypan blue dye exclusion test was used to determine the proliferation of CCRF-SB. Cells (2 × 10^5^ cells/mL) were inoculated into a 12-well plate and exposed to IMB (0, 10, 20, 30, 40, or 50 µg/mL). Then, cells were treated for 24–120 h. Trypan blue (Sigma; St Louis, MO, USA) staining was performed every day and the number of viable cells was counted using a hemocytometer.

### 2.8. Colony Formation Assay

A colony formation assay was performed as previously described [16]. CCRF-SB cells were seeded at a density of 1 × 10^4^ cells per well in 12-well plates and treated with IMB (0 or 15 µg/mL). Then, colony-forming cells were evaluated microscopically 14 days after plating. The cells were imaged with an Olympus CX31 microscope (Olympus Corporation, Tokyo, Japan) at 400× magnification. Representative images were captured using Images Plus 2.0 software (Motic Co., Ltd., Xiamen, China).

### 2.9. Giemsa Staining

Giemsa staining was performed as previously described [16]. CCRF-SB cells were seeded at a density of 2 × 10^5^ cells per well in 12-well plates and treated with IMB (40 µg/mL) for 5 days. Then, cells were harvested and fixed for 5 min in methanol, air-dried, and stained for 15 m with Giemsa stain (Sigma, 48900-500ML-F, St. Louis, MO, USA). Then, cells were rinsed with deionized water and air-dried. The morphology of the cells was observed under a light microscope (Olympus Corporation, Tokyo, Japan) at 400× magnification. Representative images were captured using Images Plus 2.0 software (Motic Co., Ltd., Xiamen, China).

### 2.10. Cell Cycle Analysis

CCRF-SB cells were seeded at a density of 1 × 10^6^ cells per well in 12-well plates and treated with IMB (0, 40, or 50µg/mL) for 24 h. Cells were harvested, washed twice with 1 × PBS, and fixed in 75% ethanol for 40 m at 4 °C. After centrifugation (2000 rpm, 5 m, 21 °C), the cells were stained with PI solution (40 μg/mL of PI in PBS with 0.1% TritonX and 100 μg/mL RNase A) for 1 h at 37 °C and analyzed using flow cytometry (BD FACSAria™ Fusion Flow Cytometer, BD Biosciences).

### 2.11. Real-Time PCR

The transcription levels of CCND1, CDK4, CDKN1C, PAX5, IKZF1, GRα and TBP were measured by RT-PCR. RNA was extracted using TRIzol reagent (Favorgen, FATRR 001, Wien, Austria). PrimeScript RT reagent Kit (Takara, RR047A, Kusatsu-shi, Japan) was used for cDNA synthesis. Real-time-PCR was conducted using TOPreal qPCR PreMIX SYBR Green with low ROX (Enzynomics; Daejeon, Korea, Republic of; RT500M). The primers used for PCR were as follows:

CCND1 Forward: 5′–AACTACCTGGACCGCTTCCT-3′

CCND1 Reverse: 5′–CCACTTGAGCTTGTTCACGT-3′

CDK4 Forward: 5′–GAAACTCTGAAGCCGACCAG-3′

CDK4 Reverse: 5′–AGGCAGAGATTCGCTTGTGT-3′

CDKN1C Forward: 5′–TGCACGAGAAGGTACACTGG-3′

CDKN1C Reverse: 5′–GTGCCTTTGGCATAACCAAT-3′

PAX5 Forward: 5′–TTGCTCATCAAGGTGTCAGG-3′

PAX5 Reverse: 5′–CTGATCTCCCAGGCAAACAT-3′

IKZF1 Forward: 5′–TGTCCCAAGTTTCAGGGAAG-3′

IKZF1 Reverse: 5′–ACGCCCATTCTCTTCATCAC-3′

GRα Forward: 5′–GGCAATACCAGGTTTCAGGAACTTACA-3′

GRα Reverse: 5′–ATTTCACCATCTACTCTCCCATCACTG-3′

TBP Forward: 5′–TATAATCCCAAGCGGTTTGCTGCG-3′

TBP Reverse: 5′–AATTGTTGGTGGGTGAGCACAAGG-3′

### 2.12. Animal Studies

To test the toxicity of IMB in vitro, bone marrow cells were extracted from wildtype C57BL/6 mice and exposed to these diterpenes as indicated, followed by the MTS assays. For in vivo toxicity testing, athymic nude mice (BALB/c-nu; 6 weeks old male; Central Lab. Animal Inc., Seoul, Korea) were divided into two groups (n = 5 per group), and vehicle (DMSO) or IMB (100 mg/kg) was injected intraperitoneally for 21 days, followed by an analysis of the white blood cells, which had been stained with the CD45 antibody in the bone marrow, spleen, and peripheral blood, by flow cytometry (BD FACSAria™ Fusion Flow Cytometer, BD Biosciences). The bodyweights of the mice were measured every other day for 21 days. Hematoxylin and eosin (H&E) staining was performed, as previously described [17]. After fixing the mouse tissue using a formalin buffer (Sigma; St Louis, MO, USA), it was embedded in paraffin. Paraffin sample blocks were sectioned into 4 µM, and after the paraffin dehydration process, it was stained with H&E. H&E-stained samples were observed under a microscope at 100× magnification (Olympus Corporation, Tokyo, Japan), and representative images were captured using Images Plus 2.0 software (Motic Co., Ltd., Xiamen, China). Animal experiments conducted in this study were reviewed and approved by the Pusan National University-Institutional Animal Care and Use Committee (PNU-2021-3056).

### 2.13. Statistical Analysis

All experiments were independently repeated at least three times to confirm reproducibility. Data are presented as mean ± standard deviation (SD). Statistically significant differences were calculated using the Mann–Whitney U test or one-way analysis of variance (ANOVA) test with Tukey’s post hoc test using Microsoft Office Excel and GraphPad Prism 5 software (GraphPad Software, Inc., San Diego, CA, USA).

## 3. Results

### 3.1. Screening of Plant Extracts as an Anti-Leukemic Agent

To search for new candidates for the treatment of B-ALL, we evaluated the native plants traditionally used as medicinal herbs in Korea. This flora group consists of 52 families and 100 species and had not been previously studied for its anti-leukemic effects (Table S1). The cells were treated with the plant extracts for 48 h or 72 h, and the cytotoxicity was analyzed by trypan blue assays. Four plant extracts that induced 60% or higher apoptotic rates were selected for further studies. We measured the selective index to compare the drug efficacy of these extracts. We isolated normal murine bone marrow (BM) cells and measured the CC_50_ value after exposure to the plant extracts (Table 1). Extracts of IMB had a higher selective index value than the others [18]. In this study, we intended to further analyze the anti-leukemic effect of IMB.

### 3.2. IMB Inhibited the Cell Growth of CCRF-SB Cells

**Figure 1:**
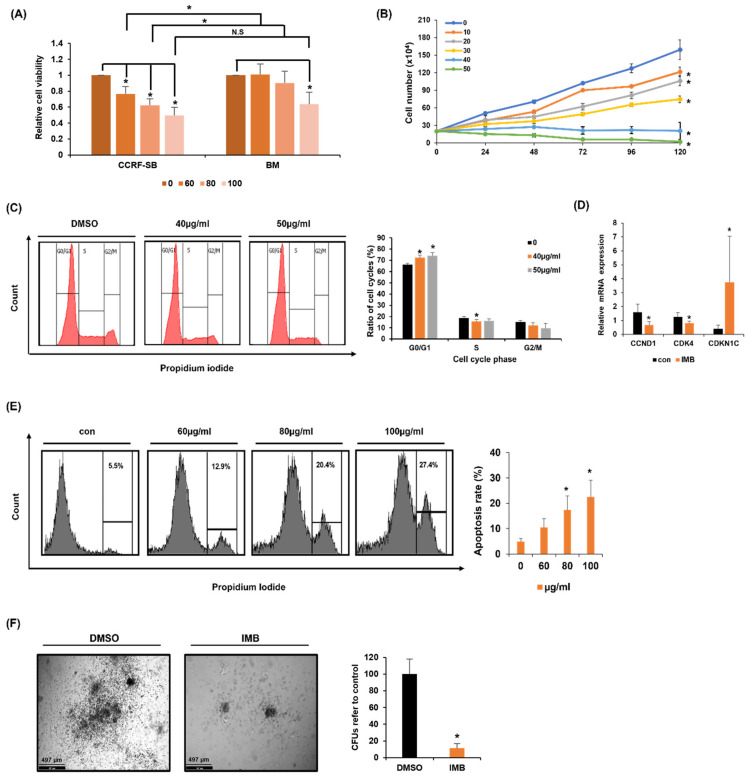
IMB inhibited cell growth in CCRF cells. (**A**) ALL cell line (CCRF-SB) or normal bone marrow cells (BM) was exposed to IMB (0, 60, 80, or 100 µg/mL) for 24 h and the MTS assay was performed to measure cell viability. N.S: not significant. (**B**) CCRF-SB cells were treated with IMB (0, 10, 20, 30, 40, or 50 µg/mL) for 120 h. Cell number was measured using the trypan blue assay. (**C**) CCRF-SB cells were treated with IMB (0, 40, or 50 µg/mL) for 24 h. The images display a representative experiment from three independent experiments. (**D**) CCRF-SB cells were cultured with IMB (50 µg/mL) for 24 h and mRNA expression of cell cycle-related genes was analyzed by RT-PCR. (**E**) CCRF-SB cells were treated with increasing concentrations of IMB (0, 60, 80, or 100 µg/mL) for 24 h, followed by PI staining, and then, the apoptotic cells were examined by flow cytometry. (**F**) To examine the effect of long-term treatment of IMB on CCRF-SB, CCRF-SB cells were treated with vehicle (DMSO) or IMB (15 µg/mL) for 2 weeks in methylcellulose and the number and size of the clones were measured. The colony images were representative of three independent experiments. Statistical significance was measured using the two-tailed Mann–Whitney test or the two-tailed one-way ANOVA test (* *p* < 0.05).

To examine the proapoptotic effect of IMB, cell viability was measured using the MTS assays. IMB had significant cytotoxicity in CCRF-SB cells in a concentration-dependent manner (Figure 1A). Intriguingly, extracts of other parts of the plant, including leaf, stalk, and fruit were not toxic to CCRF-SB cells, suggesting that components with anti-leukemic effects are present mainly in the branches of the plant (Figure S1). Additionally, we cultured CCRF-SB cells with IMB at concentrations of 10–50 µg/mL for 120 h and counted the cells every 24 h. It was confirmed that cell proliferation was inhibited dose-dependently (Figure 1B). Consistently, the fractions of G0/G1 phase cells increased, while those of S phase cells decreased (Figure 1C). To identify the genes responsible for this phenotype, the transcriptional levels of cell cycle-related genes, such as CCND1, CDK4, and CDKN1C, were analyzed by RT-PCR; mRNA levels of positive (CCND1 and CDK4) and negative (CDKN1C) regulators of cell cycle progression, were down- and upregulated, respectively (Figure 1D).

Next, to directly test the effect of IMB on the apoptotic rate of B-ALL cells, CCRF-SB cells were exposed to IMB, followed by PI-staining and flow cytometry analysis, which showed that IMB-induced apoptosis in CCRF-SB cells in a concentration-dependent manner (Figure 1E). Lastly, a methyl cellulose-based colony-forming assay was performed to confirm the inhibitory effect of long-term treatment with IMB. The number and size of the colonies decreased in the presence of IMB compared to the control (Figure 1F). In conclusion, these results suggest that IMB has an anti-leukemic effect on B-ALL cells.

### 3.3. IMB’s Antiproliferative Effect Is Cancer Cell-Specific

**Figure 2:**
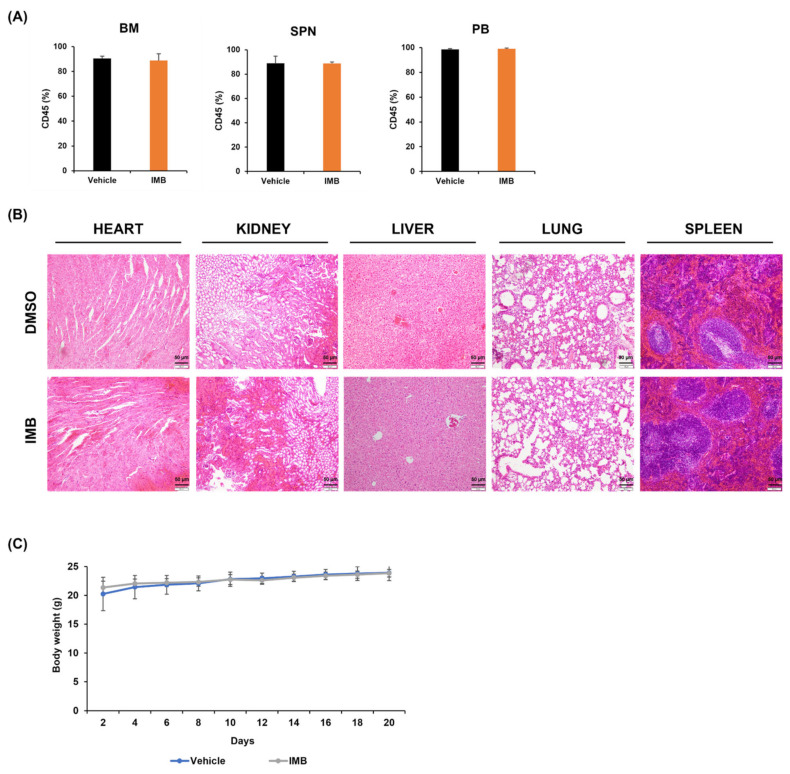
IMB has low toxicity to normal cells. (**A**) After a vehicle (100 µL of dimethyl sulfoxide, DMSO) or IMB (100 mg kg^−1^) was injected into athymic nude mice intraperitoneally daily for 21 days, the ratio of leukemic cells in bone marrow, spleen, or peripheral blood was measured by flow cytometry to confirm the toxicity of IMB to nude mice. (**B**) For histological analysis of the mouse heart, kidney, liver, lung, and spleen, H&E staining was performed. (**C**) Mouse bodyweights were measured every other day for 21 days while the injection was administered. Statistical significance was measured using the two-tailed one-way ANOVA test.

The results of the aforementioned trypan blue assays suggest that the negative effect of IMB on cell survival seems to be B-ALL cell-specific. We aimed to further investigate whether IMB has a differential effect on the apoptosis of leukemia cells and normal cells. IMB extracts were exposed to CCRF-SB cells and normal murine bone marrow cells, followed by the analysis of cell viability using the MTS assay. IMB was more toxic to CCRF-SB cells than to normal bone marrow cells in vitro (Figure 1A). Next, we tested the toxicity of IMB on normal cells in vivo. Vehicle (100 µL of dimethyl sulfoxide, DMSO) or IMB (100 mg kg^−1^) was injected into athymic nude mice intraperitoneally daily for 21 days, and bone marrow, spleen, and peripheral blood (PB) cells were harvested to measure the ratio of leukocytes by flow cytometry, which did not show any significant differences between vehicle and treatment groups (Figure 2A). To investigate the potential damage to tissues, murine heart, kidney, liver, lung, and spleen tissues were obtained and hematoxylin and eosin (H&E) staining was performed (Figure 2B). We found no morphological abnormalities under the microscope upon IMB treatment compared to control mice. In addition, we measured the body weight every other day for 3 weeks. There were no differences between the two groups, suggesting that IMB has no adverse effects on mouse physiology (Figure 2C). Together, our in vitro and in vivo data suggest that IMB’s antiproliferative effect is leukemia cell-specific.

### 3.4. IMB Induces Apoptosis by Disrupting the Mitochondrial Membrane Potential Regulated by Bcl-2 Family Members

**Figure 3:**
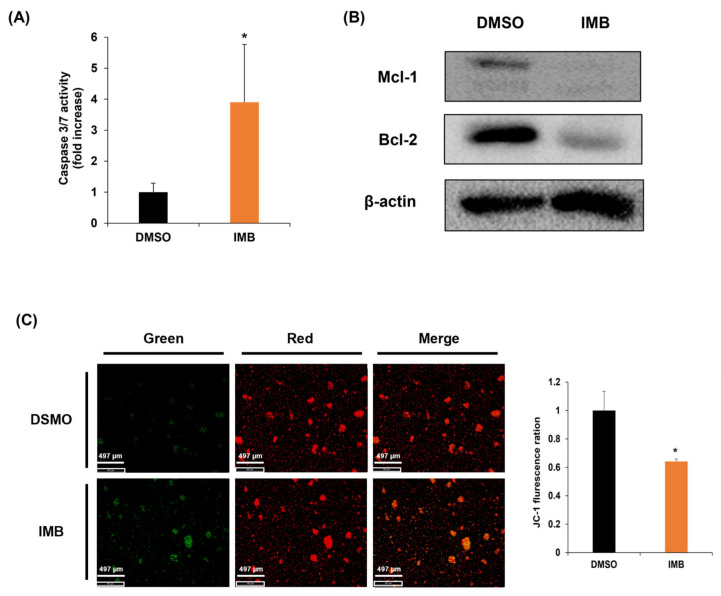
IMB reduced antiapoptotic protein expression and disrupted mitochondrial membrane potential (MMP). (**A**) The caspase-3/7 activity was observed using ELISA-based bioluminescence assays after treatment with vehicle (DMSO) or IMB (100 µg/mL) for 24 h in CCRF-SB cells. (**B**) Western blot was performed to determine the effect of IMB on the antiapoptotic proteins Mcl-1 and Bcl-2 in CCRF-SB cells. β-actin was used as a loading control. (**C**) CCRF-SB cells were cultured with vehicle (DMSO) or IMB (100 µg/mL) for 24 h and stained with JC-1, followed by observing MMP using a fluorescence microscope. Statistical significance was measured using the two-tailed Mann–Whitney test (* *p* < 0.05).

The apoptotic pathways converge on caspase activation. To test whether IMB-induced apoptosis is associated with caspase activation, we measured caspase 3/7 activity in CCRF-SB cells, following IMB treatment, which was significantly increased (Figure 3A). These results suggest that IMB induces cell death in CCRF-SB cells via the activation of caspase 3/7.

When induced by an intrinsic or extrinsic apoptotic signal, Bcl-2 family members disrupt the mitochondrial membrane potential (MMP), releasing cytochrome C. The released cytochrome C induces caspase activity, resulting in apoptosis. To investigate whether IMB-induced caspase activation and apoptosis are associated with modulating the expression of Bcl-2 family members, we analyzed the effect of IMB on the expression of antiapoptotic Bcl-2 family members, Mcl-1, and Bcl-2, which were efficiently downregulated (Figure 3B). We also performed JC-1 staining to confirm that the downregulation of these Bcl-2 family members affects the MMPs. When the membrane potential is intact, JC-1, a lipophilic, cationic dye, passes through the mitochondrial membrane and aggregates inside the membrane to emit red fluorescence. On the other hand, when the membrane potential is disrupted, it is released out of the mitochondria and becomes a monomer, emitting green fluorescence. We observed that cells incubated with IMB emitted less red fluorescence compared to controls (Figure 3C). Together, these results demonstrate that IMB disrupts MMP and increases caspase activity by reducing the expression of antiapoptotic Bcl-2 family members.

### 3.5. Upregulation of Differentiation-Related Genes by IMB Resulted in Phenotypic Differentiation of CCRF-SB Cells

**Figure 4:**
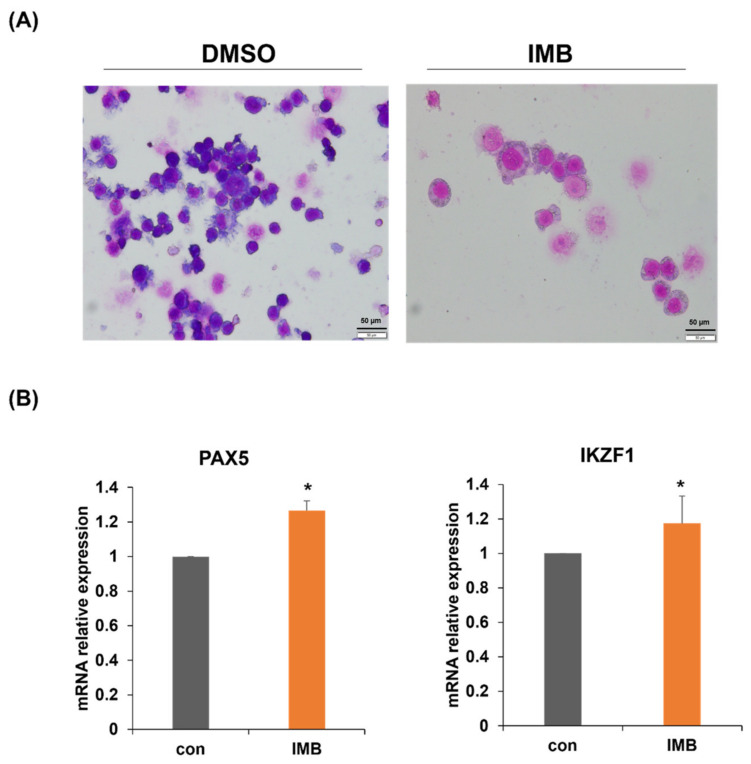
IMB affects the phenotypic differentiation of CCRF-SB cells. (**A**) CCRF-SB cells were treated with vehicle (DMSO) or IMB (40 µg/mL) for 120 h and Giemsa staining was performed. The images display a representative experiment from three independent experiments. (**B**) CCRF-SB cells were cultured with IMB (40 µg/mL) for 72 h and mRNA expression of differentiation-related genes (PAX5 and IKZF1) was analyzed by RT-PCR. Statistical significance was measured using the two-tailed Mann–Whitney test (* *p* < 0.05).

ALL is caused by the accumulation of abnormal immature white blood cells (leukemic blasts), which suggests that the promotion of differentiation, and thus, the blockade of the proliferation of these leukemia cells might be developed as a differentiation therapy. We hypothesized that IMB might inhibit cell proliferation, at least in part, by inducing differentiation. Giemsa staining showed that the nuclear/cytoplasmic ratio decreased in IMB-treated cells, which is characteristic of cell differentiation (Figure 4A). To identify the genes responsible for this phenotypic change, we analyzed the expression of PAX5 and IKZF1 at the transcriptional level, which are key regulatory genes for ALL cell differentiation (Figure 4B). PAX5 and IKZF1 mRNA expression levels were significantly upregulated after treatment with IMB for 3 days. These data and the results in the previous section imply that IMB decreases proliferation via the induction of differentiation and/or cell cycle arrest.

### 3.6. IMB Overcomes Glucocorticoid Resistance in CCRF-SB Cells by Increasing GR and Downregulating the mTOR and MAPK Signaling Pathways

**Figure 5:**
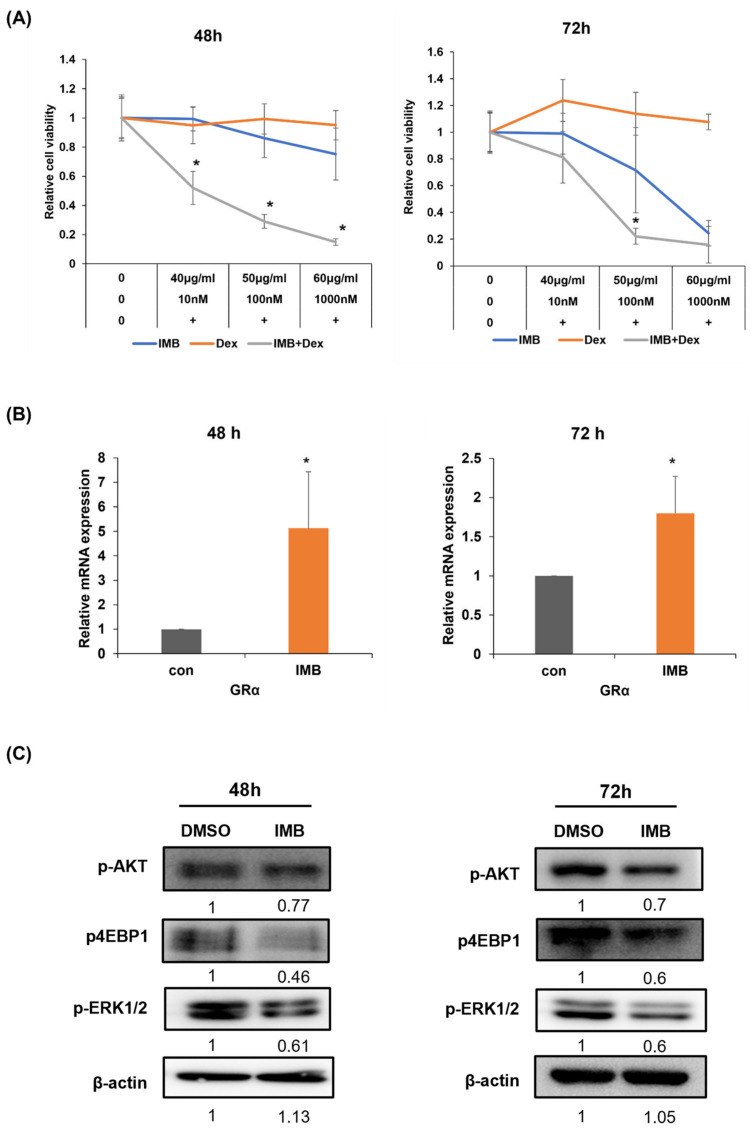
Cotreatment with IMB and dexamethasone (Dex) overcame glucocorticoid resistance. (**A**) CCRF-SB cells were exposed to IMB (0, 40, 50, or 60 µg/mL) and/or Dex (0, 10, 100, or 1000 nM) for 48 h or 72 h, and the MTS assay was performed to measure cell viability. (**B**) CCRF-SB cells were treated with IMB for 48 h or 72 h, followed by mRNA expression analysis of the glucocorticoid receptor alpha (GRα) gene by RT-PCR. (**C**) Western blotting was performed to determine the expressions of p-Akt, p-4EBP1, Bcl-2, Mcl-1, and p-Erk1/2 in CCRF-SB cells. β-actin was used as a loading control. Statistical significance was measured using the two-tailed Mann–Whitney test or the two-tailed one-way ANOVA test (* *p* < 0.05).

It has been shown that the knockdown of Mcl-1 renders ALL cells sensitive to GC, which indicates an important role for this gene in GC resistance [19]. Given our results that IMB efficiently repressed the expression of Mcl-1, we hypothesized that IMB may enhance GC-induced apoptosis in CCRF-SB ALL cells. To directly test our hypothesis, we measured the viability of CCRF-SB cells using the MTS assay after exposure to IMB and/or dexamethasone for 48 h or 72 h (Figure 5A). IMB or dexamethasone alone had no or only a limited impact on cell viability when compared to the combination treatment, which elicited a remarkable effect on cell survival, demonstrating that IMB synergizes with dexamethasone to enhance cell death in CCRF-SB ALL cells and GC resistance may be overcome by the addition of IMB. Consistent with the results was that the branches of this plant contained most of the components with anti-leukemic effects, whereas the leaf, stalk, and fruit extracts had no synergistic effect (Figure S2).

The mechanisms underlying GC resistance in ALL include the low expression of GR, which prevents GC signal from affecting intracellular signaling [7,8]. Furthermore, overexpression of the genes involved in the mTOR and MAPK signaling pathways may induce GC resistance [7,8]. GRα mRNA levels were significantly increased upon treatment with IMB (Figure 5B). A recent study revealed that PAX5 and IKZF1 regulate GRα expression and based on our results IMB increases the transcriptional levels of PAX5 and IKZF1, meaning IMB is likely to modulate GRα levels by affecting the expression of these genes. To gain further insight into the underlying mechanism through which IMB restores GC sensitivity, we examined phosphorylation levels of AKT, 4EBP1, and Erk1/2, whose hyperactivation is known to be associated with GC resistance (Figure 5C). The activities of AKT, 4EBP1, and Erk1/2 were inhibited upon treatment with IMB for 48 h and 72 h. Given that combinatory treatment of either PI3K inhibitor wortmannin or mTOR inhibitor rapamycin with dexamethasone recapitulated the synergistic effect of IMB with dexamethasone (Figure S3), we postulate that IMB overcomes GC resistance, at least partly, by inhibiting the activity of AKT and/or 4EBP1 in CCRF-SB cells [20,21,22]. Taken together, these results suggest that IMB may be used as a novel agent to restore GC sensitivity in ALL cells.

## 4. Discussion

In search of natural products with anti-leukemic effects, 100 plant extracts were screened in vitro to select novel drug candidates for the treatment of B-ALL. IMB showed the most potent effect on CCRF-SB B-ALL cells, while having much less impact on normal murine bone marrow cells, suggesting that this plant extract may have a minimal adverse effect on patients with this disease. Although IMB showed anti-leukemic activity in vitro, it is imperative to test its effect in vivo using murine models to better predict its safety and efficacy prior to possible clinical trials. The anti-leukemic effect of IMB extract was elicited in various ways: promoting apoptosis, growth arrest, and differentiation. Our mechanistic study revealed that IMB induces a proapoptotic effect via repression of anti-Bcl-2 family members, Mcl-1 and Bcl-2, which are associated with the collapse of MMP and, eventually, activation of the executioner caspase 3/7. Interestingly, only the branches of IMB but not the leaves, stems, or fruits reduced cell viability, indicating that most of the constituents with anticancer properties are contained in the branches. Although the properties of the pharmacological components in other parts of IMB, such as linoleic acid, linolenic acid, and idescarpin have been actively investigated, the bioactive constituents in the branches have yet to be identified. Therefore, future studies should focus on isolating potential therapeutic agents in the branches of IMB.

So far, few studies have been conducted on the differentiation therapy of B-ALL, which has been limited to observing phenotypic differentiation without uncovering the underlying mechanisms [23,24]. In this study, we attempted to understand the differentiation mechanisms along with the phenotypic changes. PAX5 and IKZF1 are key genes in B cell development. Thus, given that alteration in the expression of PAX5 and IKZF1 was shown to be one of the main causes of differentiation blockade in B-ALL, we speculate that the upregulation of PAX5 and IKZF1 by IMB treatment is associated with the differentiation of CCRF-SB B-ALL cells [25,26,27]. However, since B-ALL is classified into various subtypes according to genetic mutations, it may be essential to identify the subtype that would respond to IMB most efficiently.

GC is an essential chemotherapeutic drug used to treat B-ALL and resistance to this drug is associated with a poorer prognosis [28]. In particular, relapsed patients often accompany GC resistance, which is highly correlated with a low overall survival rate, suggesting that discovering a new treatment regimen is important to improve the prognosis and survival rates [29,30,31]. In the present study, we demonstrated that IMB rendered CCRF-SB cells sensitive to GC-induced apoptosis. Our data suggest that IMB restores GC sensitivity via multiple mechanisms, such as the increase in GR levels and down-modulation of mTOR and MAPK signals. Given that most of the patients with recurrent ALL are refractory to GC treatment, IMB may be used as a novel strategy to overcome GC resistance.

A recent study revealed that the mechanism through which the mTOR inhibitor rapamycin overcomes glucocorticoid resistance is related to epigenetic regulation. One study found that miR-331-3p was specifically upregulated when methylprednisolone (MP), a glucocorticoid drug, was cotreated with rapamycin in a GC-resistant ALL cell line. This miR-331-3p targeted MAP2K7, which is an essential component of the JNK/MAPK pathway [32]. Another study suggests that long noncoding RNA, GAS5, levels in peripheral blood mononuclear cells (PBMCs) obtained from healthy donors are associated with responsiveness to MP; lower GAS5 levels were correlated with better responsiveness to MP. Intriguingly, the GAS5 level was lowered upon the addition of rapamycin, resulting in the restoration of the responsiveness to MP [33]. Considering these previous results, we speculate that IMB may exert a synergistic impact on B-ALL cells, at least partly, via epigenetic regulation.

## 5. Conclusions

The current study demonstrates that IMB induces apoptosis via the inhibition of antiapoptotic Bcl-2 family members, disruption of MMP, and activation of caspase activity. It is worthwhile to note that IMB synergizes with dexamethasone to enhance cell death in B-ALL CCRF-SB cells, potentially providing a new therapeutic drug for the treatment of patients with relapsed/refractory B-ALL.

## Figures and Tables

**Table 1:** Differential cytotoxicity of plant extracts between normal BM cells and CCRF-SB cells.

NO	Scientific Name	CC50 (µg/mL) BM	IC50 (µg/mL) CCRF-SB	Selective Index (SI)
57	*Fatsia japonica* (Thunb.) Decne. & Planch.	97.20 ± 8.05	35.82 ± 1.54	2.71
61	*Isodon inflexus* (Thunb.) Kudo	171.20 ± 6.75	87.84 ± 6.66	1.95
72	*Ribes fasciculatum var. chinense* Maxim.	83.99 ± 0.23	134.07 ± 5.31	0.63
84	***Idesia polycarpa* Maxim.**	281.87 ± 4.05	85.58 ± 8.47	3.29

SI = (CC50 for normal bone marrow cell)/(IC50 for CCRF-SB cell line).

## Data Availability

Not applicable.

## Supplementary Materials

The following supporting information can be downloaded at: https://www.mdpi.com/article/10.3390/cimb45050257/s1, Table S1: Anti-leukemic effects of plant extracts against CCRF-SB cells; Figure S1: The leaf, stalk, or fruit extracts had no toxicity in CCRF-SB cells; Figure S2: The leaf, stalk, or fruit extracts did not overcome GC resistance in CCRF-SB; Figure S3: PI3K/AKT and mTOR signals sensitized GC-resistant cells to GC.
