# Supplementary material for: Anti-Leukemic Effects of Idesia polycarpa Maxim Branch on Human B-Cell Acute Lymphoblastic Leukemia Cells
Source: Curr Issues Mol Biol. 2023 May 4;45(5):4035–49. doi: 10.3390/cimb45050257 (PMC10217017; doi:10.3390/cimb45050257)
Supplement: Supplementary file 1 [file cimb-45-00257-s001.zip › Supplementary figures.pdf]

Supplementary Figure S1

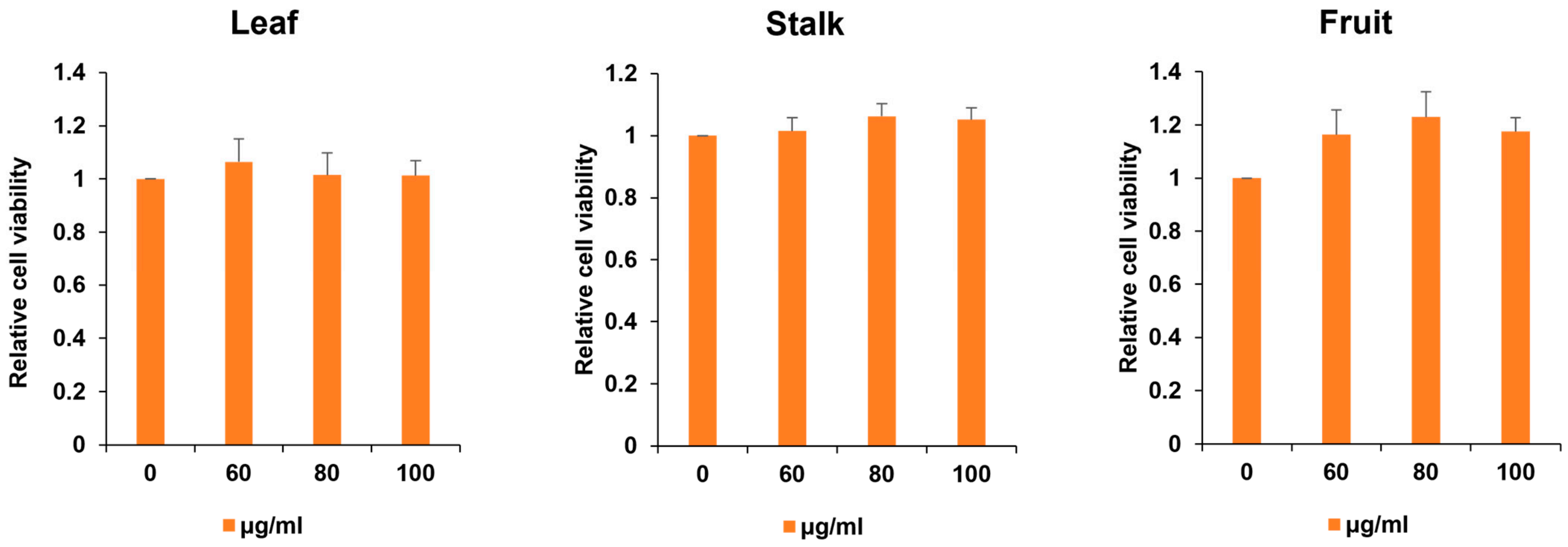

Supplementary Figure S1. The leaf, stalk, or fruit extracts had no toxicity in CCRF-SB cells.
To confirm that the leaf, stalk, or fruit extracts decrease cell viability in CCRF-SB, the MTS assay was performed. The CCRF-SB cells were cultured with the leaf, stalk, or fruit extracts (0, 60, 80, or 100 μg/ml) for 24 h. Statistical significance was measured using the two-tailed one-way ANOVA test (* $p < 0.05$).

Supplementary Figure S2

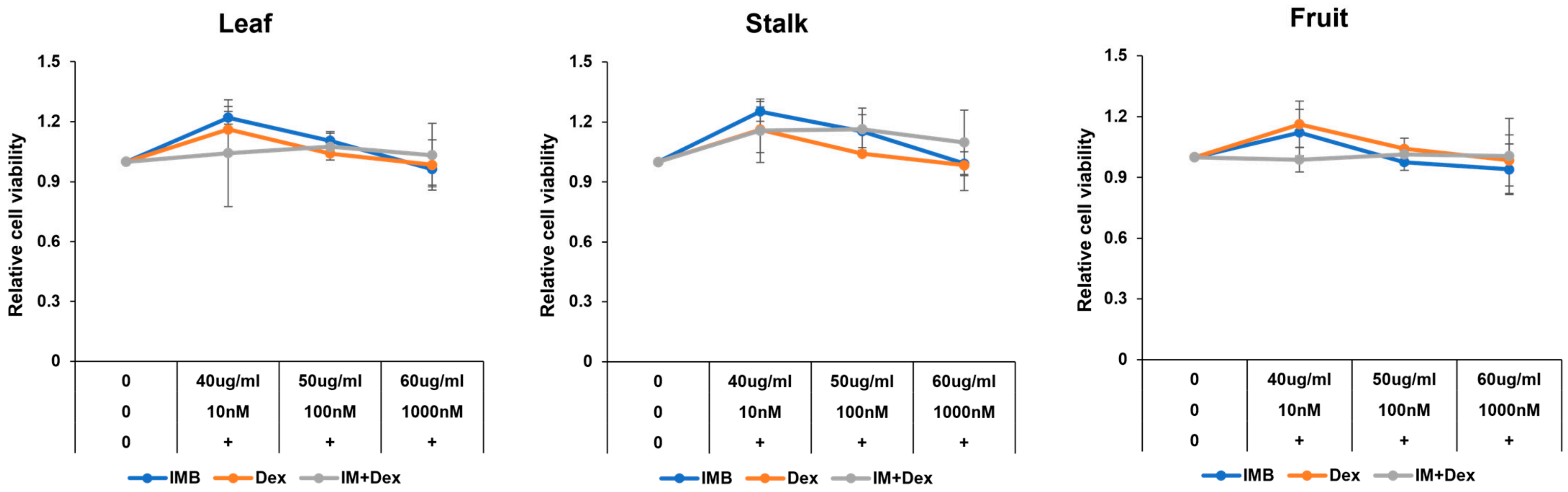

Supplementary Figure S2. The leaf, stalk, or fruit extracts did not overcome GC resistance in CCRF-SB. To test whether the leaf, stalk, or fruit extracts overcome GC resistance in CCRF-SB, the MTS assay was performed. The CCRF-SB cells were cultured with the leaf, stalk, or fruit extracts, (0, 40, 50, or 60 μg/ml) and/or Dex (0, 10, 100, or 1000 nM) for 48 h. Statistical significance was measured using the two-tailed one-way ANOVA test (* $p < 0.05$).

Supplementary Figure S3

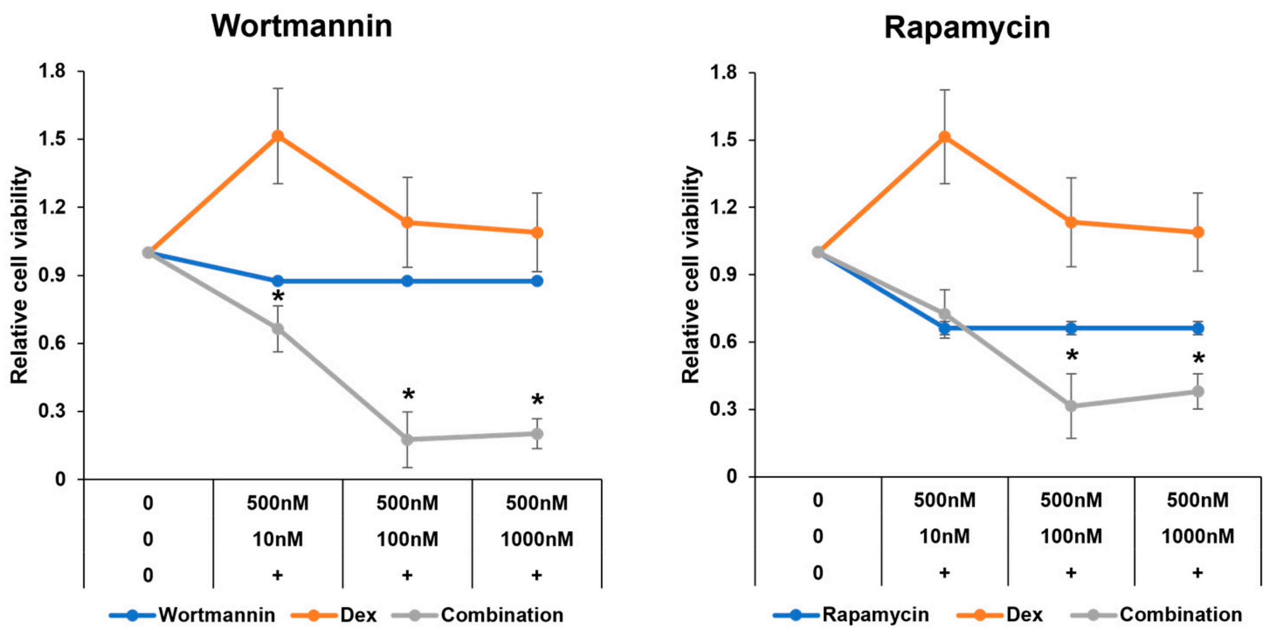

Supplementary Figure S3. PI3K/AKT and mTOR signals sensitized GC-resistant cells to GC.
To confirm that PI3K/AKT and mTOR signals have a role in overcoming GC resistance, the MTS assay was performed. The CCRF-SB cells were exposed to wortmannin (500nM) or rapamycin (500nM) in the presence/absence of Dex (0, 10, 100, or 1000nM) for 48 h. Statistical significance was measured using the two-tailed one-way ANOVA test (* $p < 0.05$).
